# Seasonal Changes in Midlife Women’S Percentage Body Fat: A 1-Year Cohort Study

**DOI:** 10.14283/jarlife.2022.4

**Published:** 2022-07-01

**Authors:** A.M. Nelson, S.L. Casperson, L. Jahns, D.G. Palmer, J.N. Roemmich

**Affiliations:** 1 USDA, Agriculture Research Service, Grand Forks Human Nutrition Research Center; 2 USDA, National Institute of Food and Agriculture

**Keywords:** Physical activity, energy intake, age, body composition

## Abstract

**Objective:**

The purpose of this longitudinal, observational study was to examine whether age and seasonal changes in sedentary activity (sedAct), moderate-to-vigorous physical activity (MVPA), and energy intake (EI) predict changes in body composition among midlife women. We hypothesized that reductions in MVPA and increases in sedAct and EI in winter, along with greater baseline age would predict increases in percentage body fat (%BF) across seasons.

**Design:**

This study used a longitudinal, within-subjects design. *Setting:* This study took place in Grand Forks, North Dakota.

**Participants:**

Participants included 52 midlife women (aged 40-60 years) who were observed over the course of one year.

**Measurements:**

Percentage body fat measures were obtained via whole body Dual Energy X-ray absorptiometry. Participants were scanned once per season. We measured EI using the ASA24®. We used a GTX3 accelerometer to measure physical activity. Each season, participants wore the monitors for 7 days, 12 hours per day. All measures began in summer.

**Results:**

Results of hierarchical multiple regression (MR) analyses showed that age increases (β = 0.310, *p* = 0.021) and summer-to-fall increases in EI (β = 0.427, *p* = 0.002) predicted seasonal increases in %BF (*R2* = .36, *F*(5, 42)= 4.66, *p* = 0.02). Changes in MVPA and sedAct were not significant predictors. Repeated measures ANCOVA revealed that summer (*M* = 37.7263, 95% CI [35.8377, 39.6149]) to winter (*M* = 38.1463, 95% CI [36.1983, 40.0942]) increases in %BF are not reversed by spring (*M* = 37.8761, 95% CI [35.9365, 39.8157]).

**Conclusions:**

To minimize increases in %BF and maintain health, midlife women, particularly older women, should be encouraged to pay extra attention to their diet in the fall months.

## Introduction

Approximately 76% of US women have overweight (body mass index (BMI) ≥ 25) or obesity (BMI ≥ 30; 1). The greatest prevalence of obesity is among women aged 40 years and older, with 43.3% classified as obese (2). Obesity is associated with greater risk for heart disease, diabetes, and some cancers (3-6). Women with obesity have a greater risk of type 2 diabetes than men (7), and the risk of diabetes increases with age (8). As such, understanding factors that contribute to greater rates of obesity in midlife women is crucial.

Age is positively correlated with weight gain in women (9, 10). This is true even when exercise remains constant (10). Weight gain is especially prevalent in women who are midlife and of menopausal age (11); however, changes in body composition (e.g., increases in body fat (12) and visceral fat (13) can occur even in the absences of weight gain (12). While this may be partly due to biological changes with aging (13), it is important to examine additional predictors that could be exacerbating midlife weight gain.

Weight gain may be more likely during certain seasons due to alterations in usual eating and physical activity. Indeed, diet, physical activity, and body weight change with season (14-16). Over the course of a year, American adults consumed more energy (kcal) per day in fall relative to spring (14). Physical activity (PA) differs across seasons as well; PA is lowest (14, 15, 17-19) and sedentary activity (sedAct) is greatest (17, 20)during winter relative to the other seasons. Weather is likely to drive physical activity changes, with conditions such as snowfall (21) and extreme weather (19) being frequently cited as barriers to engaging in physical activity. Another contributing factor to physical activity changes may be daylight; women who experience more than 14 hours of daylight engage in more moderate-to-vigorous physical activity (MVPA) than women who live in an area that is receiving less than 10 hours of daylight (22). As a result, body weight is greatest in winter (14). A study of Mexican American women reported similar findings; women gained the most weight in fall (15). Fall-winter weight gain presents a risk for gradual increases in body weight during adulthood as weight gained is not lost during spring and summer (23).

The purpose of the present secondary analysis of a longitudinal, observational study was to examine whether age and changes in sedAct, MVPA, and energy intake (EI) across seasons predict changes in body composition among midlife women. We hypothesized that reductions in MVPA and increases in sedAct, and EI in winter, along with greater baseline age would predict increases in %BF from summer to spring. As secondary aims, we investigated the changes in each of the predictor variables across seasons. As these data were derived in a location where the spring months can be as intemperate as winter months, we hypothesized that there would be greater levels of EI and sedAct in winter and spring relative to summer and fall. We also hypothesized that MVPA would be lower in winter and spring relative to summer and fall.

## Methods

### Participants

The study was completed by a total of 52 ambulatory women ranging in age from 40-60 years as previously reported (16, 24-26). Women were non-overweight, overweight, or obese as classified by BMI ranging from 18-35 kg/m2. Most women were menopausal at the beginning of the study (N = 27), as measured by follicle-stimulating hormone (FSH) levels of 25.8 mIU/ mL or greater, with 5 additional participants reaching menopausal status by winter. FSH measurement methods have been previously described (25, 26).

Participants were required to have stable weight, defined as fluctuation not exceeding ±4.5 kg for at least 6 months prior to the beginning of the study. Women were excluded from the study if they were smokers, pregnant or lactating, or had health conditions that would limit their physical activity. Furthermore, women who took medications that could potentially influence weight/ appetite were excluded. Participants were asked to refrain from engaging in intentional changes in diet or physical activity while the study was in progress.

Participants were recruited from the Grand Forks, North Dakota area through advertising throughout the community. This study was reviewed and approved by the University of North Dakota Institutional Review Board and registered with ClinicalTrials.gov(#NCT01674296). Informed consent was documented prior to the beginning of the study.

### Materials and Measures

#### Body Composition

Percentage body fat (%BF) was estimated with whole body Dual Energy X-ray Absorptiometry (DXA, GE Lunar, Madison, WI, enCORE Software Version 13.60.033). The instrument was calibrated before each session using the manufacturer’s calibration phantom. For the 248 calibrations over the 21 months of the study, mean calibration %BF was 60.53% with a standard deviation of 0.01 and a coefficient of variation of 0.016% body fat. All calibration results were within the tolerance limits recommended by the manufacturer. Participants were scanned wearing light clothing or scrubs. Analysis was conducted using iDXA proprietary software.

#### Energy Intake

EI, defined as mean calories reported consumed across each season, was derived using the National Cancer Institute’s Automated Self-Administered 24-hour Dietary Recall (27). The ASA24® is an online measure in which participants self-report the food that they have consumed over the last 24 hours, including all meals, snacks, and drinks. From these data, outcomes such as total intake of energy, carbohydrates, fat, and protein are calculated. Participants completed the ASA24® 36 times throughout the study, each completion spaced approximately 10 days apart.

#### Physical Activity

We measured physical activity using a GTX3 accelerometer (ActiGraph Corp., Pensacola, FL USA). Each season, participants wore the monitors at the hip for 7 consecutive days, 12 hours per day. Data were cleaned to remove non-wearing data (i.e., periods during which consecutive zeros were recorded for 20 min). Epochs of 15s were used for data collection. From this, we calculated total minutes of sedAct and MVPA for each season using the Crouter, Kuffel (28) algorithm and Freedson cut-points (29).

### Procedures

The present study had two cohorts; the first began in July of 2012, the second began in July of 2013. Participants visited the Research Center weekly. For the purposes of this study, we defined seasons as summer (June, July, August), fall (September, October, November), winter (December, January, February), and spring (March, April, May). All visits were conducted in the middle month of each season (i.e., July, October, January, and April).

While the visits to the Research Center were otherwise identical, the participants’ very first visit, Day 0 of the summer visit, had two unique components that occurred only at the first visit: (1) participants signed an informed consent document and (2) participants were trained during this visit on how to wear accelerometers.

On Day 0 of each season, participants came to the Research Center after a 12 h fast to complete the DXA body scan, along with other tests including completing a series of online questionnaires. The additional Day 0 tests are described in previous publications (24, 25). Before leaving the Research Center, participants were given their physical activity monitors and instructed to wear them 12 hours per day for the following 7 days. On Day 8, participants returned the physical activity monitors to the Research Center.

### Statistical Analysis

To determine whether there were changes in %BF across seasons, a multivariate repeated measures analysis of covariance (ANCOVA) was conducted with season as the repeated measure and age as the continuous covariate. Due to a violation of the sphericity assumption of compound symmetry, we used Greenhouse-Geisser corrected p values in the ANCOVA tables. For pairwise multiple comparisons of least squares means of age by season, we used Tukey-Kramer adjusted *p* values. We calculated difference scores for %BF, EI, sedAct, and MVPA between summer and fall, winter, and spring. We then mean-centered these scores for regression analyses. A significance level of ^∝^ = 0.05 was chosen a priori to determine significant *p* values. We used SAS 9.4 TS1M7 for these analyses.

We used 3 hierarchical multiple regression/correlation (MRC) models to predict summer to spring Δ%BF. In each model, age was placed in Step 1 to serve as the variable to be controlled because it was a salient predictor of Δ%BF, and all other predictors were placed in Step

2. Model 1 measured summer-fall difference scores for sedAct, MVPA, and EI. Model 2 measured summer-winter difference scores for sedAct, MVPA, and EI. Model 3 measured summer-spring difference scores for sedAct, MVPA, and EI. As secondary analyses, we used repeated measures ANOVA to investigate whether there were seasonal changes in sedAct and MVPA; one person was excluded from this analysis due to incomplete data. We used bivariate correlational analyses to further assess the relationships between the predictors and Δ%BF across seasons, as well as FSH and %BF/Δ%BF. We used SPSS Version 27.0 for these analyses.

## Results

### Primary Outcomes

There was a significant season by age interaction for %BF, *F*(1.93, 94.53) = 4.09, *p* = .021. No pairwise comparisons were statistically significant. Most (60%) participants experienced increases in %BF from summer (*M* = 37.7263, 95% CI [35.8377, 39.6149]) to winter (*M* = 38.1463, 95% CI [36.1983, 40.0942]). Summer to winter increases in %BF (Δ*M* = 0.4200, adjusted 95% CI [-0.1669, 1.0069]) were not reversed by spring (Δ*M* = -0.2702, adjusted 95% CI [-0.7754, 0.2350]). These %BF increases were not reversed by spring in 30% of the sample that were younger than the mean age of 49 years (i.e., 3 of the 10 people younger than 49 years who gained %BF,), whereas 37% the women who were older than 49 years (i.e., 7 of the 19 people over the age of 49 who gained %BF from summer to winter) did not reverse increases in %BF by the following spring. Age in the between-subjects table yielded a test statistic of *F*(1, 49) =3.77, *p* = .058. See Table 1 for means and standard deviations and Table 2 for frequencies of season-to-season changes for primary predictors.

**Table 1:** Means and Standard Deviations for Regression Predictors

Variable	M (SD)
Age (y)	49.44 (5.82)
**Energy (kcal)**	
Summer	1941.23 (428.02)
Fall	1935.34 (487.22)
Winter	1940.13 (445.17)
Spring	1900.82 (450.71)
**SedAct (min/week)**	
Summer	1022.17 (91.72)
Fall	1023.65 (83.62)
Winter	1050.61 (92.48)
Spring	1037.50 (82.14)
**MVPA (min/week)**	
Summer	44.33 (21.85)
Fall	43.81 (27.13)
Winter	37.24 (26.66)
Spring	41.31 (27.65)
**% Body Fat**	
Summer	37.77 (6.75)
Fall	37.88 (6.93)
Winter	38.15 (7.12)
Spring	37.85 (7.13)

Note: Age represents baseline age.

**Table 2:** Frequencies for Seasonal Changes in Primary Predictors

Variable	Summer-Fall		Fall-Winter		Winter-Spring	
	D/NC	I	D/NC	I	D/NC	I
EI	33	19	30	22	37	15
SedAct	41	11	37	14	45	6
MVPA	31	21	38	13	24	27

Note: D/NC represents the number of participants whose measured units decreased or did not change from season to season. I represents the number of participants whose measured units increased at least 5% from season to season.

Hierarchical MRC model 1 predicted Δ%BF, *R2* = .25, *F*(4, 47) = 3.89, *p* = .008. Age was a significant predictor in Step 1, R2 = .12, *F*(1, 50) = 6.90, *p* = .011. Adding seasonal changes in sedAct, MVPA, and EI from summer to fall [ΔR2 = .13, *Fchange*(3, 47) = 2.66, *p* = .059] did not increase the model’s ability to predict Δ%BF. In the context of the full model, increases in age and increases in EI had significant unique contributions in predicting increased %BF in spring. See Table 3 for coefficients.

Model 2 also predicted Δ%BF, *R2* = .26, *F*(4, 46) = 3.93, *p* = .008. Age was a significant predictor in Step 1, *R2* = .14, *F*(1, 49) = 7.99, *p* = .007. However, adding seasonal changes in sedAct, MVPA, and EI from summer to winter did not improve the model, *ΔR2* = .12, *Fchange*(3, 46) = 2.36, *p* = .084. In the context of the full model, age was the only significant predictor of Δ%BF. See Table 3 for coefficients.

**Table 3:** Coeficients for Hierarchical MRC Models Predicting Changes in %BF

	**Step**	**Variables**	**B**	*β*	*t*	*p*	**pr2**	**sr2**	**95% CI for B**
Summer to Fall Changes									
1		Age	.128	.348	2.63	.011*	.348	.348	.030, .226
2		Age	.101	.274	2.11	.040*	.294	.267	.005, .197
		sedAct	.002	.067	0.46	.650	.066	.058	-.008, .012.
		MVPA	-.014	-.111	0.76	.454	-.110	-.096	-.051, .023
		EI	.002	.335	2.62	.012*	.357	.331	.001, .004
Summer to Winter Changes									
1		Age	.134	.374	2.83	.007**	.374	.374	.039, .229
2		Age	.119	.332	2.43	.019*	.337	.309	.020, .218
		sedAct	.008	.269	1.86	.069	.265	.237	-.001, .017
		MVPA	.009	.058	0.38	.705	.056	.048	-.037, .054
		EI	.002	.231	1.78	.082	.254	.257	.000, .004
Summer to Spring Changes									
1		Age	.128	.348	2.62	.011*	.348	.348	.030, .226
2		Age	.121	.328	2.45	.018*	.337	.325	.022, .219
		sedAct	.000	.006	0.37	.970	.005	.005	-.009, .010
		MVPA	-.014	-.107	0.73	.469	-.106	-.097	-.052, .024
		EI	.001	.194	1.37	.176	.196	.182	-.001, .003

Note: *p < .05, **p < .01

Model 3 did not yield a better fit than the reduced model (age as only predictor) for Δ%BF, *R2* = .18, *F*(4, 47) = 2.51, *p* = .054. Though age was a significant predictor in Step 1, *R2* = .12, *F*(1, 50) = 6.90, *p* = .011, adding seasonal changes in sedAct, MVPA, and EI from summer to spring did not significantly improve the model, *ΔR2* = .10, *Fchange*(3, 47) = 1.05, *p* = .382. See Table 3 for coefficients.

### Secondary Outcomes

Though they were not found to predict Δ%BF, we investigated whether there were seasonal changes in sedAct and MVPA. We found that sedAct changed across seasons [*F*(2.55, 127.62) = 5.48, *p* = .003], increasing during winter relative to summer, *F*(1, 50) = 9.56, *p* = .003. Likewise, we found changes in MVPA across seasons [*F*(3, 150) = 4.74, p = .004]; MVPA in winter was lower than summer MVPA, *F*(1, 50) = 15.52, *p* < .001.

Results of bivariate correlational analyses revealed that summer-fall increases in EI were associated with sustained increases in %BF into both Winter [(50) = .37, *p* = .009] and spring, *r*(50) = .37, *p* = .007. SedAct in summer, fall, and winter were positively correlated with %BF in spring (*ps* < .01). There was no relationship between %BF and FSH levels seasonally, nor in the change in %BF and change in FSH levels across seasons.

## Discussion

This one-year, longitudinal study examined predictors of changes in %BF of midlife women from summer to the following spring. While we hypothesized that age and changes in EI, sedAct, and MVPA would all predict %BF changes, the most important predictors of increases in %BF from summer to spring were greater age and increases in EI from summer to fall. The magnitude of yearly increases in %BF were greater in the older women, consistent with past research (9, 10). Likewise, we found that greater EI in fall relative to summer was associated with greater increases in %BF, again consistent with past research (14, 15, 17-19). These findings suggest that while %BF increases with age, it is exacerbated by greater EI, especially during the fall. Notably, for the group as a whole there were no increases in EI from summer to fall, yet those women who reported the greatest increases in EI from summer to fall had the greatest increases in %BF during this period. (30, 31)Humans may experience similar circannual weight gain patterns to hibernating mammals; storing fat in fall seasons in preparation for food shortage in winter (30, 31). As such, excess EI in fall may be more likely to be stored as excess body fat. To our knowledge, there has not yet been work done to study this hypothesis. Though the mechanism behind the relationship between EI and %BF remains unclear, these data suggest that limiting seasonal increases in EI, especially in older women, during the fall is important because not all of the weight gained may be lost the following spring leading to the gradual weight gain with age (23).

Though not significant predictors of body composition changes, secondary analyses showed that sedAct and MVPA differed across seasons, with sedAct being greatest and MVPA being lowest in winter relative to summer. This is consistent with past research that shows people have the lowest PA and greatest sedentary behavior during winter months (14, 15, 17-20). Given the health concerns of sedentary activity (e.g., increased risk of cardiovascular disease, cancer, and diabetes; 32) and health benefits of physical activity independent of weight management (e.g., greater cardiorespiratory fitness is associated with decreased risk of mortality regardless of BMI; 33) health benefits would likely occur by limiting increases in sedAct and increasing MVPA during winter months. Contrary to our hypothesis seasonal changes in MVPA did not predict changes in %BF. Depending on the season, the women in our study engaged in 37 to 44 min/week of MVPA, far below the recommended amount of 150 min/week (34). The small magnitude of absolute MVPA limited the ability for energy expenditure to have an impact on %BF of the women in our study and the finite range of seasonal change in MVPA reduced its ability to predict change in %BF.

Strengths of the current study include the use of accelerometers to measure PA, DEXA to assess the primary outcome, %BF, and collecting three dietary recalls per month to estimate usual EI during each season. Retention was high, with only 2 women dropping out, and compliance was high for completion of PA and EI study tasks.

This study has limitations as well. The study lasted 9 months, and while it represents all four seasons over the course of one year, it is unknown whether participants would have modified EI or PA or reduced %BF if followed through a second summer. The sample is small and specialized regarding age and gender, which limits generalizability but does provide evidence for a group at risk of gaining excess adipose tissue (12, 13). The study is also limited in that the sample was from northern North Dakota, USA, an area which has great changes in both weather and sunlight throughout the year, and therefore not applicable to areas with long, hot summers and cool winters, or places where it is temperate year-round.

## Conclusions

Overall, the results of this study suggest that as women age, attention should be given to achieving or maintaining appropriate energy intake and exercise during the fall and winter months to reduce increases in %BF. Limiting increases in sedentary behavior and energy intake during the fall and winter may help women reduce seasonal increases in %BF.
